# Self-reported difficulty in walking 400 meters: the “red flag” for probable sarcopenia

**DOI:** 10.1186/s12877-022-03231-z

**Published:** 2022-06-28

**Authors:** Sara Salini, Andrea Russo, Riccardo Calvani, Marcello Covino, Anna Maria Martone, Matteo Tosato, Francesco Paolo Damiano, Anna Picca, Emanuele Marzetti, Francesco Landi

**Affiliations:** Fondazione Policlinico Universitario “Agostino Gemelli” IRCCS, 00168 Rome, Italy

**Keywords:** Muscle strength, Physical performance, Sarcopenia, Frailty, 400 m walking test

## Abstract

**Background:**

Sarcopenia is associated with adverse outcomes in older people. Several tools are recommended to assess muscle mass, muscle strength and physical performance, but are not always available in daily practice.

**Objective:**

The aim of the present study is to evaluate if there is a correlation between the personal perception of physical performance (assessed through a question on personal functional status) and the effective presence of sarcopenia (according to the EWGSOP2 definition) using data from the Longevity Check-up 7 + project.

**Design:**

Cross-sectional study.

**Setting:**

The Longevity Check-up 7 + project is an ongoing study started in June 2015 and conducted in unconventional settings (i.e., exhibitions, malls, and health promotion campaigns).

**Subjects:**

Candidate participants are eligible for enrollment if they are at least 18 years of age and provide written informed consent. For the present study subjects 65 years age old and older have been considered (*n* = 2901).

**Methods:**

According to the most recent EWGSOP2 consensus definition, subjects were defined to be affected by probable sarcopenia when handgrip strength was less than 27 kg in male and less than 16 kg in female, respectively. Furthermore, a single question assessed the perceived health status regarding own physical performance: “Do you have any difficulty in walking 400 m?”.

**Results:**

Using the EWGSOP2 algorithm, 529 (18,9%) participants were identified as affected by probable sarcopenia with a significant higher prevalence among subjects with self-reported difficulty in walking 400 m compared to participant without any difficulty (33.6% versus 13.1%, respectively; *p* < 0.001). Relative to participants without self-reported difficulty, those subjects with self-reported difficulty in walking 400 m showed a significantly higher risk of sarcopenia (odds ratio [OR]: 3.34; 95% confidence interval [CI]: 2.75–4.07).

**Conclusions:**

A single “Red Flag” question such as “*Do you have any difficulty in walking 400 m?*” should be considered as a recommended method for screening probable sarcopenia risk.

## Introduction

Sarcopenia is a geriatric syndrome characterized by an age-related loss of muscle mass, strength, and performance [1]. This age-related disorder is closely dependent on other conditions typical of modern lifestyles, such as sedentary living and inadequate or improper dietary intake. Sarcopenia will increase by 38% among older people by 2025 [2] and it is now well known that this condition has a strong impact on public health and socio-health resources [3, 4].

In particular, sarcopenia is frequently associated with a number of complications that are very common in geriatric patients such as non-self-sufficiency, reduced quality of life, falls, fractures, depression, and increased risk of hospitalization [5–7]. All these evidences, after more than thirty years from the first definition coined by Rosenberg [8], have resulted in the identification of sarcopenia as a geriatric syndrome coded as a muscular pathology with its own ICD-10 MC Diagnosis Code [9].

The recognition of sarcopenia as a real geriatric syndrome, the implications in terms of public health and the disabilities resulting from this pathology, implies a further challenge in the early identification in terms of screening in the general population. The tools currently available are devices that although simple to use (Grip Strength, Chair Stand Test) are not always available in the different health care settings. For this reason, the interest of the scientific community is to search for easier and more universally reproducible tools able to early identify the subjects at higher risk of sarcopenia in order to refer suspected cases to second level examinations and to adopt timely strategies of primary prevention [10, 11]. In this context, self-assessment tools to be submitted to the patient at the time of the screening visit are available and some of these are validated. For example, the use of the SARC F [12] self-assessment questionnaire for the identification of suspected sarcopenia cases is recommended by the EWGSOP consensus [10].

The aim of the present study is to provide whether a single question regarding the self-reported difficulty in walking 400 m correlates with the diagnosis of probable sarcopenia (according to the new EWGSOP2 definition) using data from the Longevity Check-up 7 + project.

## Materials and methods

We used the Longevity Check-up 7 + (Look-up 7 +) database, collected by an initiative of the Geriatric Medicine Department of Catholic University of Rome, planned with the aim of encouraging a healthy lifestyle in the general population. Subjects entering public spaces, such as exhibitions and/or shopping centers, or subjects adhering to specific prevention campaigns (i.e., Mese del Cuore) have been assessed using a specific questionnaire exploring lifestyle and performed a brief check-up.

The Look-up 7 + study protocol has been described in detail elsewhere [13]. Participants were considered eligible for the check-up if they were at least 18 years old and provided written informed consent. Exclusion criteria were self-reported pregnancy, blood capillary check refusal or an inability to give written informed consent. Within the context of the National Campaigns for cardiovascular prevention the Catholic University of Sacred Heart Ethical Committee ratified the entire study protocol [13].

### Study Sample

Between June 1^st^, 2015 and October 30^th^, 2021, we enrolled 13,515 individuals in different Italian cities adhering this national campaign, named “Longevity Check-up 7 + ” (Look-up 7 +) promoted by the Catholic University of Rome. For the present study, we selected all the subject 65 years age old and older. After excluding 395 subjects for missing values in the variables of interest, a sample of 2901 subjects was considered.

### Data collection

All participants who accepted to be screened underwent individual assessment comprising of a brief questionnaire, the measurement of objective cardiovascular health metrics, and the evaluation of anthropometric parameters and functional performance (lower extremity muscle power). In particular, the Look-up 7 + visit was structured to collect the following information and data: informed consent, lifestyle interview (smoking, eating habits, physical activity, previous screening performed), blood pressure measurement, weight and height assessment, and cholesterol and glucose measurements [13].

Smoking habit was categorized as current or never/former smoker. Participants who claimed to have quit smoking were still classified as smokers. Body weight was measured through an analogue medical scale. Body height was measured using a standard stadiometer. Body mass index (BMI) was defined as weight (kilograms) divided by the square of height (meters). Healthy diet was considered as the consumption of at least three portions of fruit and/or vegetables per day [13, 14]. Regular participation in physical activity was considered as involvement in exercise training at least twice a week. Cholesterol was measured from capillary blood samples using changing reagent strips based on a reflectometric system with the portable device MultiCare-In [15]. Random blood glucose was measured from capillary blood samples using changing reagent strips based on an amperometric system with the portable device MultiCare-In [15]. Blood pressure was measured according to recommendations from international guidelines [16].

### Sarcopenia definition

Muscle strength was assessed by handgrip strength, which was measured by using a dynamometer (North Coast Hydraulic Hand Dynamometer; North Coast Medical, Inc, Morgan Hill, CA). Participants performed one familiarization trial and one measurement trial with each hand, and the result from the stronger side was used for the analyses [17].

According to the most recent EWGSOP2 consensus definition [10], low muscle strength is considered as the primary domain of sarcopenia. Sarcopenia is probable when low muscle strength is detected. Hence, subjects were defined to be affected by probable sarcopenia when handgrip strength was less than 27 kg in male and less than 16 kg in female, respectively [10].

Furthermore, a single question assessed the perceived health status regarding own physical performance: “*Do you have any difficulty in walking 400 m?*” The possible answers were “Yes” or “No”. Considering that 400 m is quite difficult to quantify, practical references were made during the interview (i.e., walk to the grocery store near the house or walk the equivalent of a lap of the athletic track).

### Statistical analyses

Continuous variables were expressed as mean ± standard deviation (SD), categorical variables as frequencies by absolute value and percentage (%) of the total. Descriptive statistics were used to describe demographic and key clinical characteristics of the study population according to the self-reported difficulty in walking 400 m. The differences in proportions and the means of covariates between subjects with and without difficulty were assessed using Fisher’s Exact Test and *t* test statistics, respectively.

Cox proportional hazard models with robust variance estimates were used to assess the association between self-reported difficulty in walking 400 m and sarcopenia diagnosis. Candidate variables to be included in the Cox model were selected on the basis of biological and clinical plausibility of risk factor for sarcopenia. To identify the association between self-reported difficulty in walking 400 m and sarcopenia, we first estimated crude prevalence odds ratio and its 95% confidence interval (CI) and then controlling for age and gender (Model 1). Furthermore, multivariable Cox models were computed including age, gender, smoking habit, healthy diet, physical activity (Model 2) and age, gender, smoking habit, healthy diet, physical activity, BMI, hypertension, cholesterol level, and diabetes (Model 3).

Finally, to explore the power of self-reported difficulty in walking 400 m (independent variables) for predicting sarcopenia status (dependent variable), receiver operator characteristic (ROC) curves were separately plotted for male and female, and the area under the curve (AUC) reported, along with sensitivity and specificity at the hand grip threshold identified by EWGSOP2 for both male (27 kg) and female (16 kg).

All analyses were performed using SPSS software (version 11.0, SPSS Inc., Chicago, IL).

## Results

Mean age of 2901 subjects participating in the Longevity check-up 7 + survey was 72.7 (standard deviation 5.7, range from 65 to 98 years) years, and 1599 (55%) were women. Characteristics of the study population according to the self-reported physical performance difficulties are summarized in Table 1. More than 28% (*n* = 834) of study sample declared to have some difficulty in walking 400 m. Compared with participants without difficulty, those with self-reported difficulty in walking 400 m were significantly older, had higher BMI, and greater prevalence of hypertension and diabetes. Conversely, subjects without self-reported difficulty in walking 400 m had higher prevalence of healthy diet and were more physically active.

**Table 1:** Characteristics of study population (65 year and older) according self-reported difficulty in walking 400 m^a^

Characteristics	Total Sample (*n* = 2901)	Self-reported difficulty NO (*n* = 2067)	Self-reported difficulty YES (*n* = 834)	*p*
**Age** (years)	72.7 ± 5.7	72.0 ± 5.3	74.5 ± 6.1	< 0.001
**Gender** (Female)	1599 (55)	1047 (51)	552 (66)	< 0.001
**Smoking**	385 (13)	290 (14)	95 (12)	0.03
**Healthy diet**	2199 (76)	1606 (78)	593 (71)	< 0.001
**Physical activity**	1438 (50)	1199 (58)	239 (29)	< 0.001
**BMI** (Kg/m^2^)	26.2 ± 4.0	25.6 ± 3.5	27.8 ± 4.6	< 0.001
**Hypertension**	2267 (80)	1577 (78)	690 (84)	< 0.001
**Cholesterol** (mg/dL)	199 ± 33	201 ± 33	196 ± 31	0.01
**Diabetes**	382 (13)	235 (11)	147 (18)	< 0.001
**Sarcopenia**	529 (19)	264 (13)	265 (34)	< 0.001

Regular physical activity: physical exercise at least twice a week

Healthy diet: consumption of at least three portions of fruit and/or vegetables per day

*BMI* Body mass index

^a^Data are given as number (percent) for gender, smoking, healthy diet, physical activity, hypertension, diabetes and sarcopenia; for all the other variables, means ± SD are reported

**Fig. 1:**
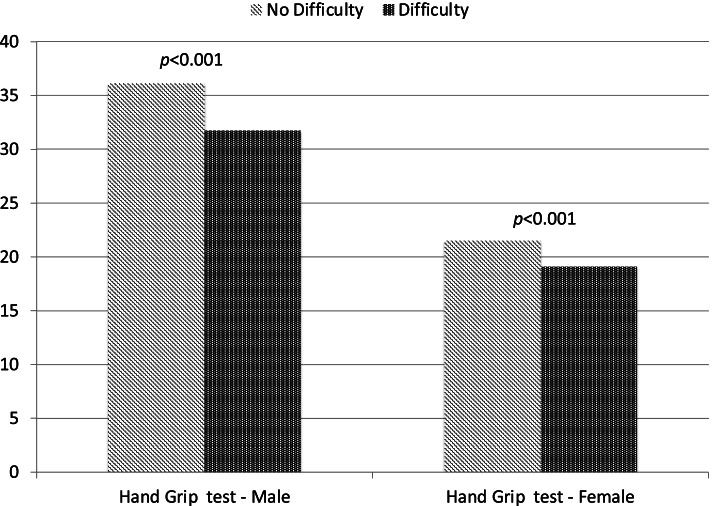
Hand grip strength according to the presence of self-reported difficulty in walking 400 m and gender

Figure 1 shows the hand grip strength test according to the self-reported difficulty in walking 400 m and gender. Subjects with self-reported difficulty had a significant lower strength compared to participants without difficulty (male: 31.7 versus 36.1, *p* < 0.001; female: 19.1 versus 21.5, *p* < 0.001).

Using the EWGSOP2 algorithm [10], 529 (18,9%) participants were identified as affected by probable sarcopenia with a significant higher prevalence among subjects with self-reported difficulty in walking 400 m compared to participant without any difficulty (33.6% versus 13.1%, respectively; *p* < 0.001). Relative to participants without self-reported difficulty, those subjects with self-reported difficulty in walking 400 m showed a significantly higher risk of sarcopenia (odds ratio [OR]: 3.34; 95% confidence interval [CI]: 2.75–4.07) (Table 2).

**Table 2:** Unadjusted and adjusted association (OR and 95% CI) between self-reported difficulty in walking 400 m and sarcopenia (defined by EWGSOP2 criteria)

	***Univariate Odds Ratio*** (95% CI)	***Adjusted*** ***Odds Ratio Model 1*** (95% CI)	***Adjusted*** ***Odds Ratio Model 2*** (95% CI)	***Adjusted*** ***Odds Ratio Model 3*** (95% CI)
**400 m difficulty**				
NO	1.0 (Referent)	1.0 (Referent)	1.0 (Referent)	1.0 (Referent)
YES	3.34 (2.75–4.07)	2.45 (1.98–3.03)	2.24 (1.79–2.80)	2.13 (1.68–2.69)

**Model 1:** adjusted for age and gender

**Model 2:** adjusted for age, gender, smoking habit, healthy diet and physical activity

**Model 3:** adjusted for age, gender, smoking habit, healthy diet, physical activity, BMI, hypertension, cholesterol level and diabetes

Estimates derived from the fully adjusted model also indicated that after adjusting for age, gender, smoking habit, healthy diet, physical activity, BMI, hypertension, cholesterol level and diabetes, participants with difficulty in walking 400 m were over two times more likely to have sarcopenia relative to individuals without difficulty and such measure of effect resulted statistically significant (OR 2.13, 95% CI: 1.68–2.69).

**Fig. 2:**
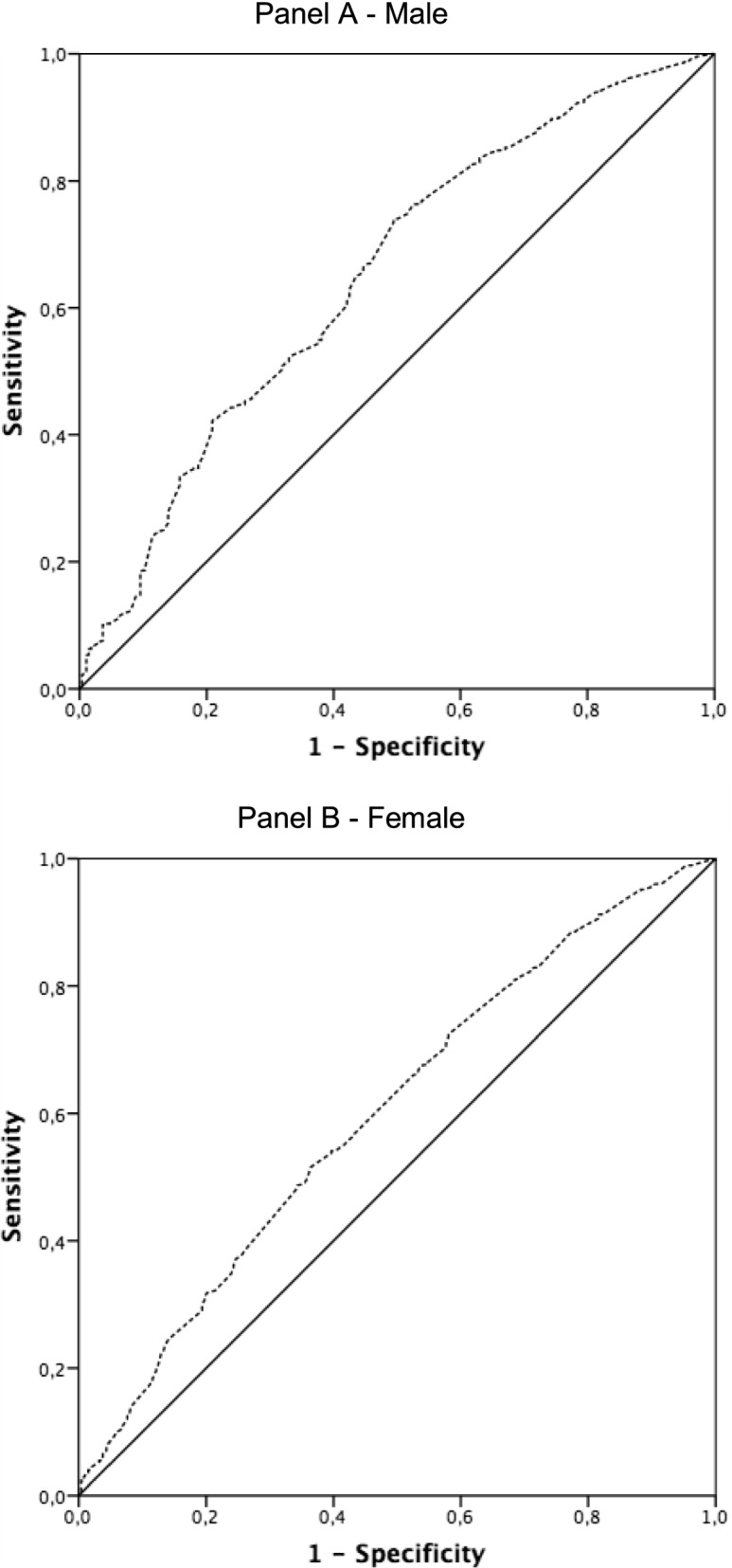
Receiver operating characteristic (ROC) curve analysis for predicting sarcopenia (by means low handgrip strength) according to the self-reported difficulty in walking 400 m. The ROC curve analysis revealed that the areas under the curves (AUCs) were 0.65 for male (Panel **A**) and 0.61 for female (Panel **B**)

**Fig. 3:**
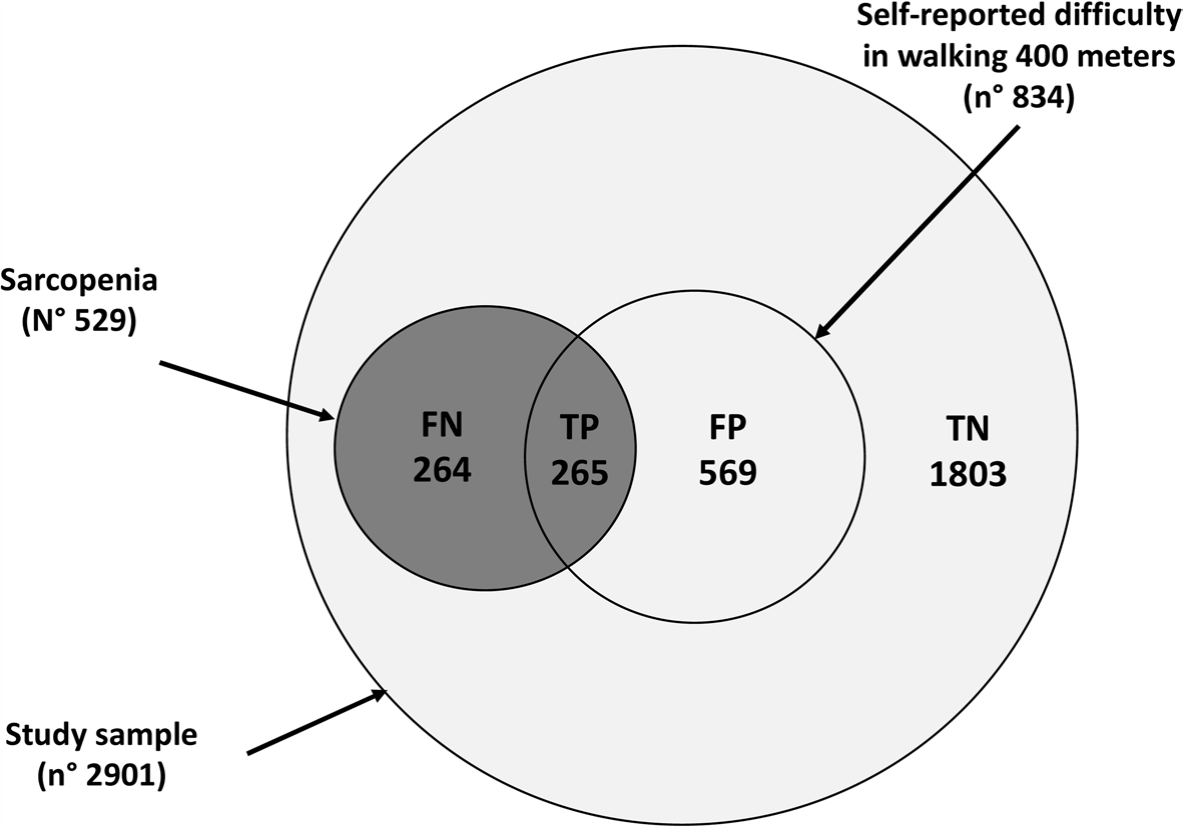
Venn diagram representation of the questionnaire test results (TP True-positive; FP False-positive; FN False-negative; TN True-negative)

The ROC curve analysis revealed that the areas under the curves (AUCs) for the self-reported difficulty in walking 400 m (independent variable) in predicting sarcopenia status (dependent variable) were 0.65 and 0.61 for male and female, respectively (Fig. 2). Considering the sarcopenia cut-off value of 27 kg in male, the sensitivity and specificity were 88% and 73%, respectively. Similarly, considering the cut-off value of 16 kg in female, the sensitivity and specificity were 88% and 77%, respectively. Furthermore, to better understand how many subjects complained of difficulty walking did not have possible sarcopenia a Venn diagram of possible sarcopenia and difficulty walking has been produced (Fig. 3). Accordingly, the negative predictive value was 88.7 [87.7 – 89.6] and positive predictive value was 24.0 [21.8 – 26.4].

## Discussion

Our study shows that there is a correlation between the perception of the own physical performance and the diagnosis of probable sarcopenia. In particular, subjects who declared to have difficulty in walking 400 m were twice as likely to be affected by sarcopenia compared to those who did not report any difficulties. Subjects who declared to have difficulties in walking 400 m were also older, with higher BMI and with more frequent findings of altered values of glycemia and cholesterol. Participants who did not have difficulties in walking 400 m showed a higher rate of healthy diet and physical activity level. Furthermore, using the ROC analysis the present results clearly indicate that self-reported difficulty in walking 400 m significantly correlated to the presence of probable sarcopenia, evaluated according to the recent EWGSOP2 consensus [10]. Overall, these results suggest that it is possible to consider the single question “*Do you have any difficulty in walking 400 m?*” as a useful tool (*red flag*) to identify subjects at higher risk of sarcopenia.

In 2019, the EWGSOP2 consensus [10] published the latest indications for the diagnosis of sarcopenia with particular emphasis on early identification, defining as probable sarcopenia the alteration of muscle strength detected by handgrip strength and/or chair stand test. Several instruments are currently available for measuring muscle mass, muscle strength and physical performance, making it possible to better characterized the sarcopenia status. In particular, DXA, BIA, CT and MRI are recommended for the measurement of muscle quantity and quality; handgrip strength and chair stand test for the measurement of muscle strength and gait speed, 400 m walking test and Short Physical Performance Battery (SPPB) for the measurement of physical performance. However, for many different reasons – health care professional education, time to perform the assessment, equipment availability, costs – these instruments are not always implemented in clinical practice.

In 2016, Beaudart and colleagues [18] proposed to early identify the patients at risk of sarcopenia by promoting the “*Red Flag Model*”. The model is based on the identification of screening tools, comprising the combination of clinician's observation and assessment of specific signs and symptoms, which would make possible to identify and select patients for a second-level examinations and eventually prevention/treatment interventions. Furthermore, some validated questionnaires, such as the Mini Sarcopenia Risk Assessment (MSRA) [19] and SARC F [20], have been suggested to be useful to identify subjects at risk of or affected by sarcopenia. SARC-F is the extensively recommended and suitable tool for community screening sarcopenia comprising 5 questions that investigate the ability to lift weights, walk, stand up from a chair, climb stairs and the number of referred falls in the last year [20]. Another simple and used test is the Mini Sarcopenia Risk Assessment (MSRA) Questionnaire [19], a 7-item survey assessing the following aspects: age, protein and dairy products intake, daily meals, physical activity level, number of hospitalizations and weight loss in the last year. Other similar assessments have also been developed with the same purpose, such as Taiwan Risk Score for Sarcopenia [21], Sarcopenia Scoring Assessment Model (SarSA-Mod) [22], and SAR-QoL questionnaire [23].

Even thought, all these sarcopenia screening tests have been validated—often on very small samples and selected populations—and are fairly quick to perform, none of these are based on a single item (*reg flag*). The use of a questionnaire consisting of a single and easy question has advantages in terms of quicker administration by the operator, larger number of screened subjects, easier to implement in all clinical settings (especially in the GP's office) and greater compliance by the respondent. In this respect it is important to highlight that the present study is the first attempting to determine how a single item is sufficiently sensitive and specific to screen for sarcopenia among old subjects in community.

The choice of question “*Do you have any difficulty in walking 400 m?*” was based on scientific evidence showing that the ability to walk 400 m represents the cut-off for identifying those subjects at greater risk or with physical disabilities. The inability to complete the 400-m walking test within 15 min without sitting, leaning, or the help of another person is included in the EWGSOP2 algorithm as a criteria for assessing the sarcopenia severity [10]. Similarly, the ESCEO Experts group [24] includes the 400-m walking test among the inclusion criteria to be fulfilled for phase II and III clinical pharmacological trials for sarcopenia. Furthermore, the 400-m walking test was also used as the main outcome in the two largest and most important trials—Life Study [25] and SPRINT-T Study [26]—which evaluated the impact of physical exercise on the prevention of incident physical disability.

Albeit dealing with a highly relevant issue, our study presents several limitations that need to be discussed. First, because the study is cross-sectional, it is difficult to identify a causal relationship between the response to the questionnaire and the diagnosis of probable sarcopenia, made by assessing muscle strength. A deeper understanding of the correlation between self-reported difficulty in walking 400 m and sarcopenia requires the analysis of prospective data that are not available at this stage for our study. Second, the type of screening activities could have partially influenced the results. For example, even though the sarcopenia assessment was performed according to standard protocols, people who decided to participate in the study procedures were involved — before being assessed — in usual activities, such as walking, carrying bags, and eating. The activities, performed immediately before being evaluated, could have influenced the assessment. Third, this type of screening does not provide access to other key information, such as the presence of arthritis, osteoporosis and/or other musculoskeletal and neurological diseases that have a direct impact on muscle strength and physical performance assessment, and also on the perception of one's health status and functional capacity. However, for the type of participants recruited in the study, it is possible to exclude that acute illnesses were present at the time of evaluation. Data collection always took place in conjunction with parallel sporting or preventive health events, which required a reasonable autonomy of the participants. Considering the type of evaluation setting, specific muscle mass and muscle quality data – BIA and/or DXA – are not available to confirm the diagnosis of probable sarcopenia according to the EWGSOP2 consensus [10]. Finally, the population included only Caucasian persons, so these results may not be applicable to other ethnic groups.

Apart from these limitations, this study offers a unique opportunity to investigate the relationship between the single question “*Do you have any difficulty in walking 400 m?”* and the presence of a probable sarcopenia. The results clearly show that self-reported difficulty in walking 400 m was positively associated with sarcopenia identified according to the EWGSOP2 criteria [10]. Given the negative health implications of sarcopenia [27, 28], its early identification using an easy and fast screening question in the general population represents a new challenge, considering the importance of maintaining psychophysical well-being and satisfactory social and relational life, as well as preventing physical disability and its consequences. Results from this study, with a low positive predictive value, imply that false positives still need to undergo further diagnostic testing. However, although some possible cases of sarcopenia might be missed (false negative), the high negative predictive value may allow in a population-based screening to exclude all negative tests from further diagnostic testing.

In conclusion, based on this observation, the self-reported difficulty in walking 400 m could be suggested as a method for the early detection of individuals at risk of probable sarcopenia. Simplifying the screening process using a single question could help to earlier identify sarcopenia and consequently to be more proactive in implementing prevention and treatment strategies [29, 30]. Furthermore, in the near future the use of technological systems (i.e., smartphones, social media) could help to reach a greater segment of the population and enhance the usefulness of this tool.

## Data Availability

The datasets used and/or analysed during the current study available from the corresponding author on reasonable request**.**
